# City limits: Heat tolerance is influenced by body size and hydration state in an urban ant community

**DOI:** 10.1002/ece3.6247

**Published:** 2020-04-15

**Authors:** Dustin J. Johnson, Zachary R. Stahlschmidt

**Affiliations:** ^1^ Department of Biological Sciences University of the Pacific Stockton California

**Keywords:** critical temperature, knock‐down, thermal maximum, urban heat island, water availability

## Abstract

Cities are rapidly expanding, and global warming is intensified in urban environments due to the urban heat island effect. Therefore, urban animals may be particularly susceptible to warming associated with ongoing climate change. We used a comparative and manipulative approach to test three related hypotheses about the determinants of heat tolerance or critical thermal maximum (*CT*_max_) in urban ants—specifically, that (a) body size, (b) hydration status, and (c) chosen microenvironments influence *CT*_max_. We further tested a fourth hypothesis that native species are particularly physiologically vulnerable in urban environments. We manipulated water access and determined *CT*_max_ for 11 species common to cities in California's Central Valley that exhibit nearly 300‐fold variation in body size. There was a moderate phylogenetic signal influencing *CT*_max_, and inter (but not intra) specific variation in body size influenced *CT*_max_ where larger species had higher *CT*_max_. The sensitivity of ants’ *CT*_max_ to water availability exhibited species‐specific thresholds where short‐term water limitation (8 hr) reduced *CT*_max_ and body water content in some species while longer‐term water limitation (32 hr) was required to reduce these traits in other species. However, *CT*_max_ was not related to the temperatures chosen by ants during activity. Further, we found support for our fourth hypothesis because *CT*_max_ and estimates of thermal safety margin in native species were more sensitive to water availability relative to non‐native species. In sum, we provide evidence of links between heat tolerance and water availability, which will become critically important in an increasingly warm, dry, and urbanized world that others have shown may be selecting for smaller (not larger) body size.

## Graphical abstract

Larger urban ant species had greater heat tolerance (critical thermal maxima, *CT*_max_). Water availability increased *CT*_max_ in an urban ant community. *CT*_max_ and thermal safety margins in native species were more sensitive to water availability.

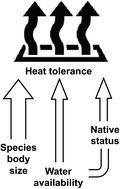

## 1 INTRODUCTION

Temperatures are increasing globally due to climate change (IPCC, 2014; Oreskes, 2004), and high temperatures may alter survival, growth, and reproduction in animals (Angilletta, 2009; Angilletta et al., 2007; Huey & Stevenson, 1979; Savage, Gillooly, Brown, West, & Charnov, 2004). The sensitivity of animals to high temperatures can be determined by features of the thermal performance curve, including the critical thermal maximum (*CT*_max_, the temperature at which an animal loses essential motor function: reviewed in Angilletta, 2009). The *CT*_max_ metric is an established proxy for assessing heat tolerance (Lutterschmidt & Hutchison, 1997) that links whole‐animal performance to organismal fitness, species’ distribution, and outcomes of interspecific interactions (Angilletta et al., 2007; Diamond, Chick, Penick, et al., 2017; Huey & Stevenson, 1979; Wiens, Graham, Moen, Smith, & Reeder, 2006). It has been used to assess heat tolerance in both invertebrates and vertebrates (Baudier, Mudd, Erickson, & O'Donnell, 2015; Geerts et al., 2015; Zhang & Kieffer, 2014) from a diversity of habitat types (e.g., aquatic, tropical, and urban environments: Diamond, Chick, Perez, Strickler, & Martin, 2017; Geerts et al., 2015; Nguyen et al., 2017). Further, it can be used to understand an animal's thermal safety margin (herein, the difference between an animal's *CT*_max_ and the maximal temperature of its environment), which is an important metric for predicting animals’ responses to ongoing climate change (Khaliq, Hof, Prinzinger, Bohning‐Gaese, & Pfenninger, 2014; Sinclair et al., 2016; Sunday et al., 2014).

Variation in heat tolerance of terrestrial animals may be driven by a range of factors. First, body size may influence *CT*_max_ variation where large body size may lead to higher *CT*_max_ perhaps due to increased heat shock protein (Hsp) synthesis and/or reduced thermal conductance of integument (Galushko et al., 2005; Gehring & Wehner, 1995; Hood & Tschinkel, 1990). On the other hand, smaller body size may be associated with higher *CT*_max_ because a smaller body size increases the relative surface area available for heat loss, and warming may select for smaller body size (e.g., temperature‐size rule and Bergmann's rule: reviewed in Angilletta, 2009; Gardner, Peters, Kearney, Joseph, & Heinsohn, 2011; but see Horne, Hirst, & Atkinson, 2015). Second, variation in *CT*_max_ may also be explained by animals’ adaptations to local microenvironments, which are changing with climate change (Stillman & Somero, 2000; Sunday et al., 2014) and may be linked to body size (Kaspari, 1993). For example, animals living in warmer microenvironments may be adapted to have higher *CT*_max_ values than those from cooler microenvironments (Baudier et al., 2015) due to variation in membrane composition, or in the production of isoenzymes or Hsps (Gabriel & Lynch, 1992; Stillman & Somero, 2000; Gabriel, Luttbeg, Sih, & Tollrian, 2005; Pincebourde & Casas, 2019; reviewed in Angilletta, 2009; Hochachka & Somero, 2002). Third, phylogeny can influence animal physiology (Cahan et al., 2017; Gutierrez‐Pesquera et al., 2016; Rezende, Bozinovic, & Garland, 2004), and closely related species may, therefore, exhibit similar *CT*_max_ values regardless of differences in morphology or microenvironment preferences. Thus, it is crucial to account for body size, local adaptation, and phylogeny when determining this important metric of thermal sensitivity.

Examining the determinants of heat tolerance in urban animals is critical because cities are rapidly expanding (Grimm et al., 2008), and global warming is intensified in urban environments due to the urban heat island effect (Andrew, Hart, Jung, Hemmings, & Terblanche, 2013; Oke, 1973; Pincebourde, Murdock, Vickers, & Sears, 2016; Youngsteadt, Dale, Terando, Dunn, & Frank, 2015). Consequently, urban environments can reduce animals’ thermal safety margins, giving animals little buffer to further increases in environmental temperature (Chown & Duffy, 2015; Diamond, Chick, Perez, et al., 2017). However, the thermal hazard of the urban heat island effect may be offset by an increased availability of water because many cities are subsidized with water, especially in warmer or more arid regions exhibiting rapid human population growth (McCarthy, Best, & Betts, 2010; McCluney, Burdine, & Frank, 2017; Vahmani & Jones, 2017). The availability of water constrains terrestrial life, and hydration state plays a critical role in *CT*_max_, body temperature, and homeostasis (Da Lage, Capy, & David, 1989; Manenti, Cunha, Sørensen, & Loeschcke, 2018; Nguyen et al., 2017; Smit et al., 2018). Desiccation can enhance the physiological heat shock response in some species (flies: Benoit et al., 2010; Gotcha, Terblanche, & Nyamukondiwa, 2018); yet, in other species, it reduces *CT*_max_ and does not increase the upregulation of inducible Hsps during a heat shock (ants: Nguyen et al., 2017). Thus, a comparative examination of the effects of body size, thermal life history, and water availability on *CT*_max_ in terrestrial animals is required, and such a comprehensive approach may also provide insight into community dynamics associated with invasion biology. For example, overlapping thermal and hygric niches explain the success of invasions by multiple species of fruit flies and the concomitant decline in a native species of fruit fly (reviewed in Duyck, David, & Quilici, 2006). Yet, the invasive Argentine ant may be particularly vulnerable to desiccation, which may limit its success in warmer, drier habitats (Schilman, Lighton, & Holway, 2007). Therefore, species‐specific variation in thermal safety margin or *CT*_max_ sensitivity to hydration may predict competition outcomes between native and non‐native species in warming urban environments.

We used two experiments to first test a set of three hypotheses related to determinants of heat tolerance within and among species—specifically, that (a) body size, (b) chosen microenvironments, and (c) hydration status influence *CT*_max_. For our first hypothesis, we predicted that larger animals would have relatively high *CT*_max_ values. Second, we predicted that animals using warmer microenvironments would have higher *CT*_max_ values because these animals regularly experience higher temperatures (sensu coadaptation of thermal physiology and thermoregulatory behavior: reviewed in Angilletta, 2009). Third, we predicted that well‐hydrated animals would have relatively high *CT*_max_ values. Although our study examined a community of ants from western North America (see below), these first three predictions are based on work in ants from other regions (Cerda & Retana, 2000; Clemencet, Cournault, Odent, & Doums, 2010: western Europe; Ribeiro, Camacho, & Navas, 2012; Baudier et al., 2015: neotropics; Nguyen et al., 2017: eastern North America; but see Hemmings & Andrew, 2017: Australia). We also tested a fourth hypothesis that native species are particularly physiologically vulnerable in urban environments. Specifically, we predicted that native species would exhibit reduced thermal safety margins and *CT*_max_ values and exhibit *CT*_max_, thermal safety margins, whole‐body water content values that are more sensitive to water availability relative to non‐native species. This prediction is based on work demonstrating that invasive species may benefit from urbanization or climate change (Buczkowski & Richmond, 2012; Lejeusne, Latchere, Petit, Rico, & Green, 2014; Menke et al., 2011; Vonshak & Gordon, 2015; Zerebecki & Sorte, 2011).

To test our hypotheses, we determined *CT*_max_ in ants common to cities in California's Central Valley after manipulating and quantifying hydration state (i.e., via water limitation and measuring animals’ water content), and accounting for variation in body size (nearly 300‐fold variation in live mass), phylogeny (11 species), and local microenvironments (surface temperatures chosen by ants during activity). Recent work comparing *CT*_max_ values in ants across urban and rural populations has improved our understanding of how urban environments influence the evolution of thermal tolerance traits (Angilletta et al., 2007; Diamond, Chick, Perez, et al., 2017; Diamond, Chick, Perez, Strickler, & Martin, 2018). However, our study used a multi‐species approach to comprehensively examine the factors influencing an important metric of heat tolerance in urban animals that may be particularly adapted for a reliance on water to reduce thermal hazards—the study area is characterized by hot, dry summers, as well as water subsidization (i.e., regular irrigation). Thus, our study offers unique insight into the role of water availability in heat tolerance across a community, which is important in an increasingly warm, dry, and urbanized world (Angilletta, 2009; Grimm et al., 2008; Oke, 1973; Pincebourde et al., 2016; Sarhadi, Ausín, Wiper, Touma, & Diffenbaugh, 2018).

## 2 MATERIALS AND METHODS

### 2.1 Research system

Ants are abundant and important components of terrestrial ecosystems (Underwood & Fisher, 2006), including urban ecosystems (e.g., Menke et al., 2011; Penick, Savage, & Dunn, 2015; Stahlschmidt & Johnson, 2018). They are effective behavioral thermoregulators and, thus, are adapted and sensitive to a wide range of temperatures (Angilletta et al., 2007; Chick, Perez, & Diamond, 2017; Jumbam, Jackson, Terblanche, McGeoch, & Chown, 2008; Lighton & Turner, 2004; Underwood & Fisher, 2006). Also, shifts in microenvironments due to climate change are expected to be particularly important to small‐bodied animals, such as ants (Hemmings & Andrew, 2017; Pincebourde et al., 2016; Pincebourde & Suppo, 2016; Scheffers, Edwards, Diesmos, Williams, & Evans, 2014).

Because populations near the edge of a species' range are expected to be at the extreme end of the environmental stress gradient (Gaston, 2009; Han et al., 2019; Magi, Semchenko, Kalamees, & Zobel, 2011; Sexton, McIntyre, Angert, & Rice, 2009), sampling such edge populations may misrepresent species‐wide thermal physiology and thereby confound comparative analyses. Therefore, the populations of all species used in the experiments were well within species’ geographical and/or latitudinal ranges (AntWeb). Ants used in the experiments (Figure S1; Table S1) were collected on sunny days in June–August in Stockton or Lodi, California, which are cities characterized by a hot‐summer Mediterranean climate (Kottek, Grieser, Beck, Rudolf, & Rubel, 2006).

### 2.2 Experiment 1

In 2017, an interspecific comparison was used to examine the effects of body size, microenvironmental temperature, and water availability on ants’ *CT*_max_ values. A total of 683 individuals from 11 species (seven native species, and four non‐native species) across 37 colonies were collected (Figure S1; Table S1). From 10:00 to 14:00, ants were collected via an aspirator along foraging trails on both impervious and nonimpervious surfaces (e.g., bare soil and concrete, respectively) in shaded and unshaded conditions as described previously (Stahlschmidt & Johnson, 2018). At each colony, six different temperature readings of ground surface were taken using an infrared thermometer (Fluke 62 MAX) at the time of sampling. To estimate the temperatures of microenvironments chosen by ants during activity (*T*_active_), three temperature readings were taken on each ant trail approximately 1 m from one another. To estimate the range of ants’ thermal options, three temperature readings were also taken near the ant trail (*T*_available_) where directionality (0–360°) and distance (1–8 m) from each ant trail were determined via a random number generator. The maximal temperature of these six readings (i.e., *T*_active_ and *T*_available_) was used to estimate each ant's thermal safety margin (i.e., the difference between its *CT*_max_ [see below] and the maximal temperature of its environment).

Collected ants were brought back to the University of the Pacific in Stockton, CA, and they were provided *ad libitum* water (water‐filled shell vials with cotton plugs) in 470 ml round glass storage containers. Granulated table sugar was provided as a food source even though mild food limitation (e.g., 1 day of starvation) does not affect thermal tolerance in other insects, including ants (Bubliy, Kristensen, Kellermann, & Loeschcke, 2012; Nguyen et al., 2017; Overgaard, Kristensen, & Sørensen, 2012). Ants were kept in these open‐top containers (i.e., unsealed containers with no lid; *n* = 1–30 ants per group replicate depending on ant body size; *n* = 2–28 group replicates per species; see Table S1 for details) overnight at room temperature (~21°C) and a 14:10 light:dark cycle, which approximates the mean summer temperature and light:dark cycle for Stockton, CA (National Weather Service). At 8:00 the next morning, ants were assigned to one of two water treatment groups: unlimited or limited access to water, where the latter treatment group had water‐filled vials replaced with empty vials until *CT*_max_ trials later in the day (see below). Preliminary trials indicated that this duration of water deprivation did not influence mortality across our study species for Experiment 1. Live body mass, *CT*_max_, dry body mass, and live water content were determined as described in *CT*_max_ *Trials* below.

### 2.3 *CT*_max_ *trials*

Starting at 15:00–16:00 (i.e., the warmest time of day in the field), ants underwent *CT*_max_ trials. Prior to each trial, the live body mass of ants was recorded. Due to limitations of the available analytical balance (±0.1 mg), ants were typically pooled together as a group replicate (e.g., five ants) and weighed in Experiment 1 to determine an average value of pretrial live mass. Then, each group replicate (*n* = 2–28 per species; see Table S1) was placed into an open‐top 236 ml round glass storage container in a 24°C water bath (note: each individual ant was weighed and then placed in a 30 ml glass container for Experiment 2; see below). An empty open‐top 236 ml (Experiment 1) or 30 ml (Experiment 2; see below) container was also placed into the water bath, and a thermocouple was attached to the floor of each empty container to estimate ant body temperature (estimated *T*_body_) in real‐time. After 30 min of acclimation, the water bath was heated and estimated *T*_body_ increased 0.5°C/min until all of the ants were knocked down. The *CT*_max_ for each ant was determined by its knock‐down temperature, which was the estimated *T*_body_ at which an ant lost the ability to right itself (mean: <50 min). Before they could recover from knock‐down, the group replicates of ants (Experiment 1) or individual ants (Experiment 2; see below) were all euthanized by placing them into a 50°C drying oven. After ≥24 hr, ants were reweighed to estimate ant body size (dry mass) and relative (%) live water content.

### 2.4 Experiment 2

To better understand how *CT*_max_ was affected by water limitation, *T*_active_, and intraspecific variation in body size, *CT*_max_ was determined in 2018 for two focal, native species: winter ant, *Prenolepis imparis* (*n* = 118 ants; *n* = 5 colonies) and field ant, *Formica moki* (*n* = 114 ants; *n* = 5 colonies). *Prenolepis imparis* is readily found throughout the contiguous United States whereas *F. moki* is found in the western United States (Sanders, Barton, & Gordon, 2001; AntWeb). The two species are fairly sympatric as both are common in wooded urban environments. Despite these similarities, results from Experiment 1 indicated that these species varied greatly in *T*_active_ and *CT*_max_, and their *CT*_max_ values responded differently to water limitation (i.e., 8 hr of water limitation reduced *CT*_max_ in *P. imparis*, but not in *F. moki*; see Section 3). Thus, examining both species allowed us to examine the roles of water limitation and intraspecific variation in body size in species with dissimilar thermal biology.

**FIGURE 1:**
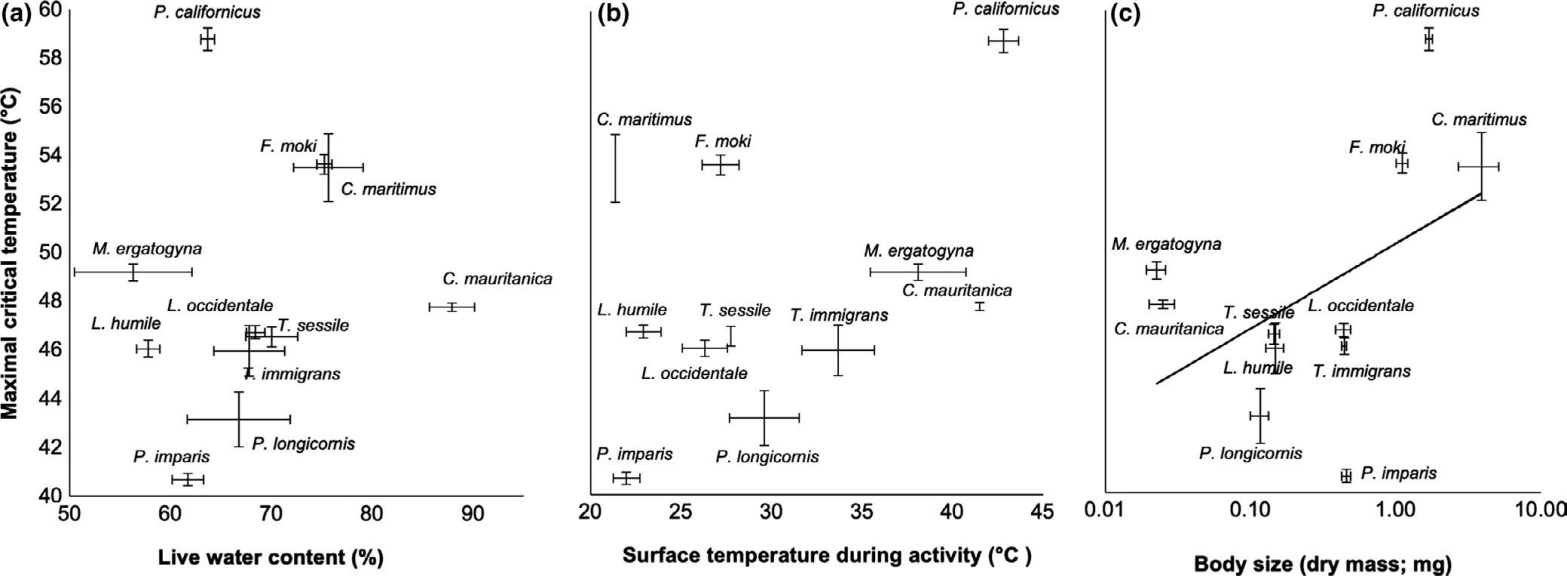
Relationships between maximal critical temperature (*CT*_max_) and (a) live water content, (b) active temperature (temperatures of microenvironments chosen during activity), and (c) body size for a community of urban ants in California's Central Valley (11 species; *n* = 683) in Experiment 1. Values are displayed as mean ± *SEM* across group replicates, and include *CT*_max_ values for data pooled across both water treatment groups (11 species; *n* = 683 ants; see text for details). As indicated by the regression line, only body size was significantly correlated with *CT*_max_ after accounting for phylogeny

**FIGURE 2:**
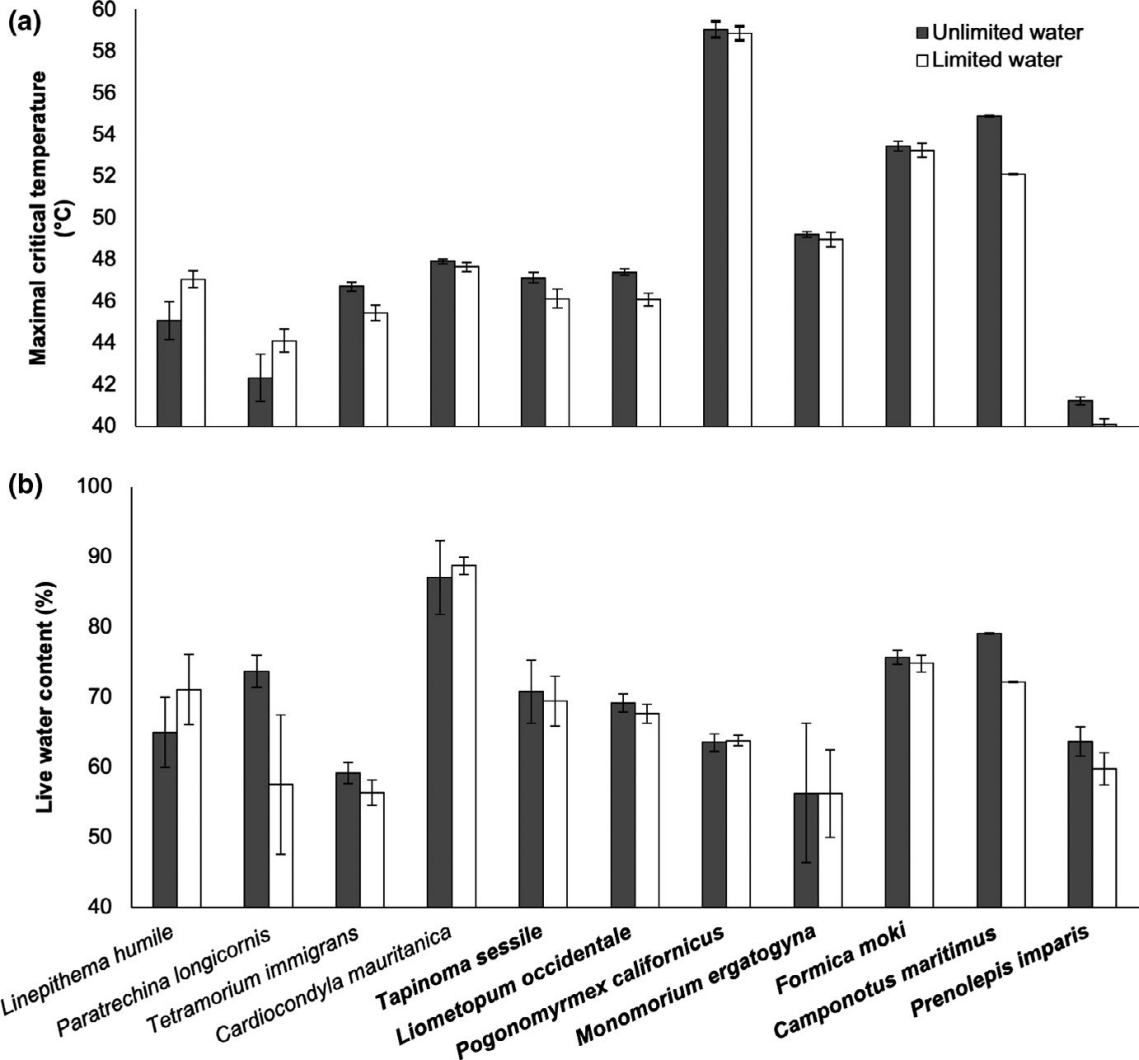
Effects of water treatment (white: 8 hr water limitation; gray: unlimited water) on (a) maximal critical temperature (*CT*_max_) and (b) live water content in a community of urban ants in California's Central Valley (*n* = 683 individuals) in Experiment 1. Values are displayed as mean ± *SEM* across individuals for *CT*_max_ and across group replicates for live water content (see text for details). Native species’ names are bolded

**FIGURE 3:**
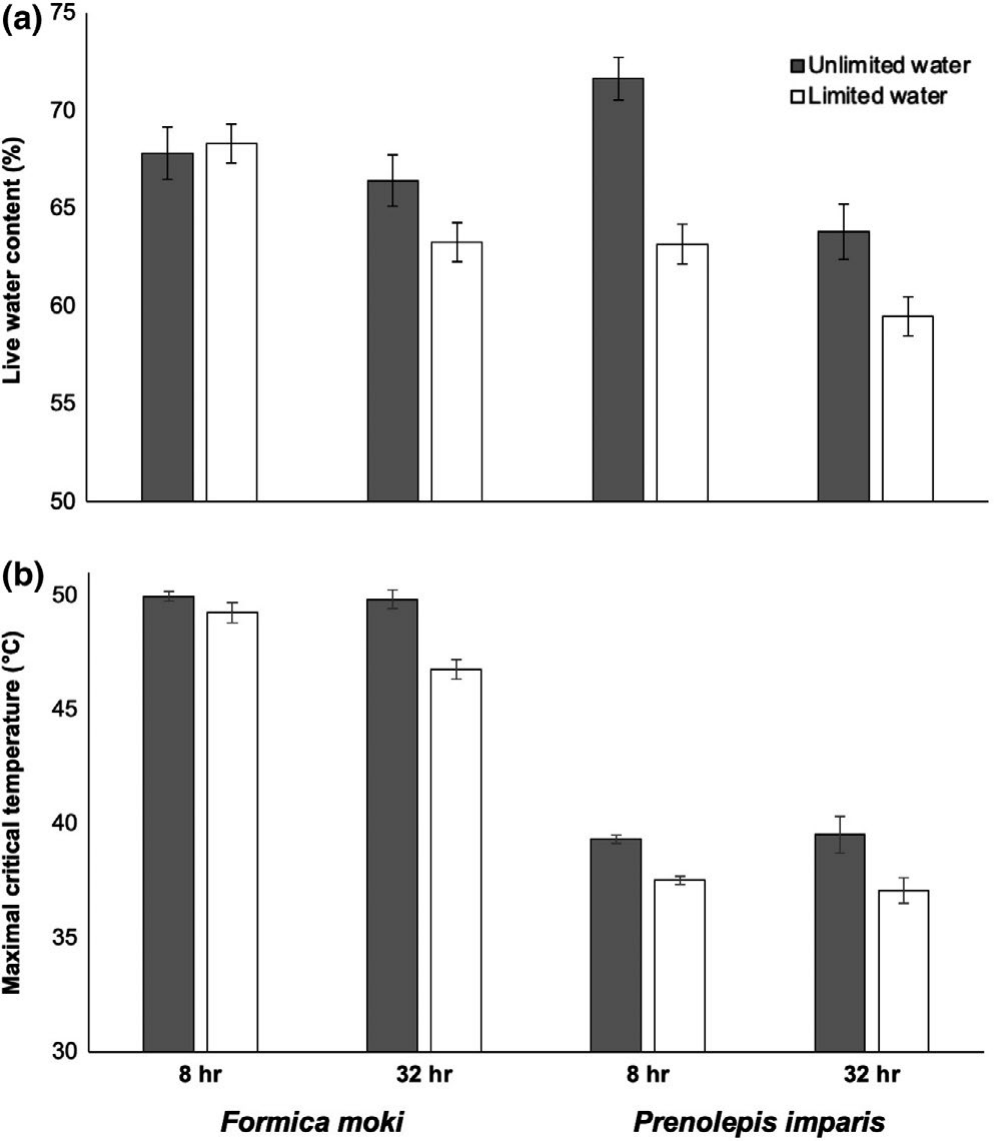
Effects of species, water treatment (white: water limitation; gray: unlimited water), and duration of water treatment on (a) live water content and (b) maximal critical temperature in two species of native urban ants (*Formica moki* and *Prenolepis imparis*) in California's Central Valley (*n* = 232 individuals) in Experiment 2. Values are displayed as estimated marginal mean ± *SEM* because body size (dry mass) and mean *T*_active_ were each included as a covariate (see text for details)

Although similar to Experiment 1, the methods of Experiment 2 were modified in three ways. First, the effect of intraspecific variation in body size on *CT*_max_ was determined because the mass of each ant could be determined (mean live mass: 2.6 mg), rather than relying on group replicate data for body mass as in Experiment 1. Second, multiple water limitation treatment levels were used (8 and 32 hr of water limitation, rather than only 8 hr in Experiment 1). Captive housing may influence *CT*_max_ independent of water availability (e.g., ants housed for 32 hr with unlimited water may exhibit different *CT*_max_ values than those housed for 8 hr with unlimited water). Therefore, water‐limited and ‐unlimited ants were assessed for *CT*_max_ at each time point to control for captive housing effects. Third, a more comprehensive estimate of *T*_active_ was achieved in Experiment 2 by taking the six temperature measurements as in Experiment 1 three times during activity (across 2 hr intervals) each sampling day, rather than just once at the time of sampling as in Experiment 1. Dependent variables (e.g., *CT*_max_ and *T*_active_) were similar across sampling years for *P. imparis* and *F. moki* (Figures 1a,b, 2, and 3).

### 2.5 Statistical analyses

To determine relationships between variables of interest (e.g., body size [dry mass] and *CT*_max_) across study taxa in Experiment 1 (i.e., to test our first set of three hypotheses), two analytical methods were used. First, a software for comparative analyses (COMPARE: ver. 4.6b, open‐access) was used to perform linear regression analyses that accounted for the effects of phylogeny. Previous work on heat tolerance indicates that using phylogenetically informed models results in consistently better fits of the data than noninformed models (Leiva, Calosi, & Verberk, 2019). Thus, *CT*_max_ was regressed on each dependent variable from Hypotheses 1–3 (dry body mass, *T*_active_, and relative [%] body water content, respectively) using three linear regression analyses (i.e., one analysis for each independent variable) and phylogenetically generalized least squares methods (PGLS). The maximum likelihood estimate of alpha, the parameter of phylogenetic dependence based on the Ornstein‐Uhlenbeck model for trait evolution, for each pair of variables was determined on a scale from 0 to 15.5 (Freckleton, Harvey, & Pagel, 2002; Hansen, Pinaar, & Orzack, 2008; Martins & Hansen, 1997). For PGLS, a low alpha (near 0) suggests data are highly dependent on phylogeny, whereas a high alpha suggests data are generally independent of phylogeny. The phylogenetic tree (Figure S1) for our study taxa included estimated minimum branch lengths and was constructed from established taxonomic sources (Janda, Folková, & Zrzavý, 2004; Moreau, Bell, Vila, Archibald, & Pierce, 2006; Ward, Brady, Fisher, & Schultz, 2015).

Second, several linear mixed models were run in SPSS (ver. 22, IBM Corp.) to test all four hypotheses in Experiment 1. In these models, data were log‐transformed when necessary (e.g., to achieve normally distributed residuals), and two‐tailed significance was determined at *α* = 0.05 using Satterthwaite approximations of *p*‐values. Species and nest identity were included as random effects in all linear mixed models on data from Experiment 1. For group replicates in Experiment 1, one model included mean *CT*_max_ of group replicates as the dependent variable to test Hypotheses 1–3. In this model, water treatment (unlimited or limited) was included as a fixed effect to test Hypothesis 3, and body size (mean dry mass of group replicates) and mean *T*_active_ (i.e., mean of three temperature readings of group replicates; see above) were included as covariates to test Hypotheses 1 and 2, respectively. That is, Hypotheses 1–3 were tested by phylogenetically informed, single‐factor models (see above), as well as by one multi‐factor mixed model that accounted for phylogeny by including species as a random effect. To test Hypothesis 4, three additional models were fit where *CT*_max_, estimated thermal safety margin, and relative (%) live water content were each included as a dependent variable. The model on relative live water content used data from mean values of group replicates while those on *CT*_max_ and estimated thermal safety margin used data from individual ants. In these models, water treatment, native status, and treatment × native interactions were included as fixed effects.

For Experiment 2, a model for *CT*_max_ in each species included water treatment (unlimited or limited), time since water treatments were assigned (8 or 32 hr), and a treatment × time interaction as fixed effects to test Hypothesis 3, and body size (dry mass) and mean *T*_active_ as covariates to test Hypotheses 1 and 2, respectively. To determine the effect of water treatments on live water content, another mixed model was run on relative (%) live water content for each species with water treatment, time, and a treatment × time interaction as fixed effects. Water availability can facilitate food intake in insects (Raubenheimer & Gade, 1994, 1996; Padda & Stahlschmidt, in revision), meaning that effects of water limitation on *CT*_max_ may have been driven by the effects of food limitation. Therefore, to determine whether water‐unlimited ants ate more food than water‐limited ants during the water‐manipulation period, a mixed model was also run on body size (dry mass, which would increase with food intake) for each species with water treatment, time, treatment × time interaction as fixed effects, and nest identity as a random effect.

## 3 RESULTS

### 3.1 Experiment 1

Comparative regression analyses (phylogenetically generalized least squares methods [PGLS]) on data from Experiment 1 indicated moderate phylogenetic dependence (alpha values ranged from 2.2 to 3.3). Results (e.g., significance levels and regression coefficients) were similar across data sets from both water treatment groups—thus, displayed data and reported results from Experiment 1 represent the entire data set (i.e., pooled across both water treatment groups). In Experiment 1, *CT*_max_ was significantly influenced by body size (Figure 1a; *F*_1,9_ = 5.6; *p* = .042; *R* ^2^: .34). However, there was not a detected effect of *T*_active_ on *CT*_max_ (Figure 1b; *F*_1,9_ = 1.6; *p* = .21; *R* ^2^: .20) or relative (%) live water content (Figure 1c; *F*_1,9_ = 0.32; *p* = .59; *R* ^2^: .028). Mixed model analysis on Experiment 1 data agreed with results from the PGLS analyses: *CT*_max_ was influenced by body size (*F*_1,100_ = 4.7, *p* = .032), but there was not a detected effect of *T*_active_ on *CT*_max_ (*F*_1,32_ = 0.56, *p* = .46). Mixed model analysis also indicated that *CT*_max_ was affected by water treatment where water limitation reduced heat tolerance (*F*_1,94_ = 4.4, *p* = .038; Figure 2a).

There was not a detected effect of native status on *CT*_max_ (*F*_1,9_ = 1.9, *p* = .21), but *CT*_max_ was influenced by the interaction between native status and water treatment (*F*_1,653_ = 6.8, *p* = .010) where *CT*_max_ in native ants was more sensitive to water availability (Figure 2a). There was not a detected effect of native status on estimated thermal safety margin (*F*_1,5_ = 4.2, *p* = .10), but estimated thermal safety margin was affected by water treatment where margins were greater for water‐unlimited individuals (*F*_1,650_ = 7.5, *p* = .0063) and a native × treatment interaction (safety margins in native ants were more sensitive to water availability; *F*_1,649_ = 5.1, *p* = .025). Relative (%) live water content was influenced by water treatment (*F*_1,92_ = 4.1, *p* = .046), but not there was not a detected effect of native status (*F*_1,6_ = 0.012, *p* = .92) or a native × treatment interaction on relative live water content (*F*_1,92_ = 0.22, *p* = .64; Figure 2b).

### 3.2 Experiment 2

In *P. imparis*, *CT*_max_ was influenced by water treatment (*F*_1,108_ = 32, *p* < .001), but there was not a detected effect on *CT*_max_ due to the time since water treatments were assigned (i.e., 8 or 32 hr; *F*_1,109_ = 0.32, *p* = .57), a time × water treatment interaction (*F*_1,108_ = 0.72, *p* = .40), *T*_active_ (*F*_1,104_ = 1.9, *p* = .18), or body size (dry mass; *F*_1,111_ = 0.33, *p* = .57; Figure 3a). The relative (%) live water content of *P. imparis* was influenced by water treatment (*F*_1,110_ = 4.0, *p* = .047) and time (*F*_1,110_ = 4.1, *p* = .045), but there was not a detected effect of a time × water treatment interaction on relative live water content (*F*_1,110_ = 0.094, *p* = .76; Figure 3b).There was not a detected effect on dry mass due to water treatment (*F*_1,110_ = 0.060, *p* = .81), the time since water treatments were assigned (*F*_1,110_ = 0.030, *p* = .86), or a time × water treatment interaction (*F*_1,110_ = 0.026, *p* = .87).

In *F. moki*, *CT*_max_ was influenced by water treatment (*F*_1,105_ = 18, *p* < .001), the time since water treatments were assigned (*F*_1,105_ = 8.2, *p* = .005), and a time × water treatment interaction (*F*_1,105_ = 6.8, *p* = .010), but there was not a detected effect of *T*_active_ (*F*_1,103_ = 0.31, *p* = .62) or body size (*F*_1,108_ = 0.65, *p* = .42) on *CT*_max_ (Figure 3a). The relative live water content of *F. moki* was influenced by water treatment (*F*_1,106_ = 13, *p* < .001) and a time × water treatment interaction (*F*_1,106_ = 6.2, *p* = .015), but there was not a detected effect of time alone on relative live water content (*F*_1,107_ = 0.25, *p* = .62; Figure 3b). Water availability did not appear to influence food intake because there was not a detected effect on dry mass due to water treatment (*F*_1,106_ = 3.0, *p* = .086), the time since water treatments were assigned (*F*_1,106_ = 0.26, *p* = .61), or a time × water treatment interaction (*F*_1,106_ = 1.3, *p* = .27).

## 4 DISCUSSION

Using a comparative and manipulative approach, we demonstrate complex dynamics of temperature sensitivity in a widespread animal taxon. Urban ant species varied in *CT*_max_ in a body size‐dependent fashion (Figure 1c). Although water availability had overall positive effects on body water content and *CT*_max_ across the ant community, these effects also varied across species (Figures 2 and 3). For example, body water content and *CT*_max_ in *P. imparis* were strongly dependent on short‐term water availability while these variables in *P. californicus* were unaffected by short‐term water availability (Figure 2). However, results from Experiment 2 indicate that body water content and *CT*_max_ can be insensitive to water limitation in the shorter‐term in some species, but not in the longer‐term (e.g., *F. moki*: Figure 3). Thus, studies focusing on individual species or those using limited experimental treatments may yield varying and/or misleading results related to understanding an eco‐physiological metric of increasing importance (Khaliq et al., 2014; Leiva et al., 2019; Sunday et al., 2014). Last, our results indicate that native ants may be more physiologically vulnerable than non‐native ants because the sensitivity of *CT*_max_ and thermal safety margins to water availability in native ants was greater than in non‐native ants (Figure 2a).

An animal's body size influences many aspects of its physiology and ecology—from egg size to population size (Peters, 1986; Savage et al., 2004; Smith & Lyons, 2013). Likewise, body size influenced *CT*_max_ across species of urban ants in support of our first hypothesis (larger animals have greater heat tolerance: Figure 1c). Similar results have been demonstrated in other ants (Cerda & Retana, 2000; Clemencet et al., 2010; Ribeiro et al., 2012; Baudier et al., 2015; but see Hemmings & Andrew, 2017; Baudier et al., 2015) and other insects (Le Lagadec, Chown, & Scholtz, 1998). This may be due to larger animals having a greater thermal inertia (Le Lagadec et al., 1998), more water stores (increased evaporative cooling potential, but see below), or greater Hsp levels (but see Brown et al., 2007; Moreno, Merino, Martinez, Sanz, & Arriero, 2002). Although larger body size may be more beneficial for heat tolerance in terrestrial animals, experimental and biogeographical evidence indicates strong selection for smaller body size due to warming (e.g., temperature‐size rule and Bergmann's rule: reviewed in Angilletta, 2009; Gardner et al., 2011; but see Horne et al., 2015). Clearly, future work is required to determine the relative magnitude of these competing selective pressures in terrestrial animals (i.e., for larger body size due to heat tolerance benefits vs. smaller size via temperature‐size rule) and the role of other factors that may mediate these pressures, such as phylogenetic constraints or local environmental variation (e.g., oxygen levels in aquatic environments: Verberk, Leuven, Velde, & Gabel, 2018). There was not an effect of intraspecific variation in body size on *CT*_max_, which agrees with other studies examining physiological variation within species (desiccation tolerance: Mogi, Miyagi, Abadi, & Syafruddin., 1996). This is likely due to greater genetic and phenotypic variation across species, rather than within species (Gearty, McClain, & Payne, 2018)—for example, we detected nearly 300‐fold variation in body mass across species in Experiment 1, but only 5‐fold variation in body mass within species in Experiment 2. Future work should examine the effect of body size on heat tolerance using a more accurate balance (e.g., ±0.01 mg or ±0.001 mg rather than ±0.1 mg as in our study) and in additional species because our study only thoroughly investigated its effect in two species (i.e., *F. moki* and *P. imparis*).

For both experiments, our second hypothesis (animals active in warmer microenvironments have higher *CT*_max_ values) was not supported. Microhabitat temperatures have been associated with heat tolerance in other ants (Baudier et al., 2015), and discrepancies between this study and our study may be due to differences in the methodologies of temperature measurement. In our study, an infrared thermometer was used to collect temperature measurements of surfaces used by ants during activity. In the study by Baudier et al. (2015), miniature temperature data loggers were used to collect measurements, which allowed for continuous temperature data collection (i.e., many temperature measurements). However, we failed to detect an effect of *T*_active_ on *CT*_max_ within two focal species even after significantly increasing the number of temperature measurements from Experiment 1 to Experiment 2. Coadaptation between thermoregulatory behavior and thermal physiology is not always supported (reviewed in Angilletta, 2009), as exemplified by our results testing for the relationship between *T*_active_ and *CT*_max_ within and among species. This behavior‐physiology mismatch may be due to an acquisition tradeoff between nutritional and thermal resources where animals are obligated to forage in suboptimal temperatures (i.e., nutritional benefits outweigh thermoregulatory costs: Andrew et al., 2013; Andrew & Terblanche, 2013).

As described above, our third hypothesis (hydration status influences *CT*_max_) was supported by mixed model analyses in Experiment 1 and Experiment 2. Our results indicate that ants have a threshold at which water limitation affects their heat tolerance, and these thresholds vary across species (Figures 2a and 3b). Other physiological metrics (e.g., cold tolerance and stress) also exhibit thresholds, and these thresholds can influence higher levels of biological organization (e.g., species distributions: reviewed in Martinez, Arenas, Trilla, Viejo, & Carreno, 2015). Therefore, it is increasingly important to understand such thresholds in the context of global climate change and urbanization. Although body water content was not significantly related to *CT*_max_ (Figure 1a), water limitation generally led to a decrease in body water content and in reduced heat tolerance (Figures 2 and 3). Therefore, water limitation in our study did not facilitate cross‐tolerance, which is when exposure to one stressor better equips an animal to tolerate a subsequent and different stressor (reviewed in Harrison, Woods, & Roberts, 2012). However, other work has shown a link between mechanisms underlying responses to desiccation and heat stress (Benoit et al., 2010; Gotcha et al., 2018). Continued work is required to better understand factors influencing contradictory results, such as those due to variation in taxon and/or methodology (e.g., life stage of desiccation exposure, or the duration of desiccation or recovery from desiccation). Related, we detected effects of water limitation on body water content and effects of interspecific variation in body size (dry mass) on heat tolerance—however, the relatively low sensitivity of the analytical balance used in our study (±0.1 mg) likely introduced error into our data and may have constrained our ability to parse finer‐scale effects on these gravimetric variables.

There are at least three general types of mechanisms that may underlie the costs of dehydration to heat tolerance. First, dehydration may confer reduced evaporative cooling potential because fewer water stores can be deployed (i.e., lost to release heat) during periods of heat stress. We indirectly assessed this mechanism in our study (see Appendix S1). Our calculations reveal that hydration likely did not confer an appreciable evaporative cooling advantage of animals in our study. Our experimental design for determining *CT*_max_ (i.e., using partially submerged glass containers in a water bath) likely reduced evaporative cooling by ants, and recent work similarly indicates that very little water is lost by other small insects during exposure to thermal ramping associated with determining *CT*_max_ (Manenti et al., 2018).

Second, desiccation or water limitation may lead to shifts in resource use or allocation patterns that result in a weaker heat stress response. For example, dehydration may reduce energy use (i.e., metabolic rate), which, in turn, reduces evaporative water lost through respiration (Marron, Markow, Kain, & Gibbs, 2003; reviewed in Chown, Sorensen, & Terblanche, 2011). Because metabolic rate and Hsp levels may be linked (Dahlhoff, Buckley, & Menge, 2001; Folguera et al., 2011; Sammut & Harrison, 2003), a reduction in metabolic rate (i.e., energy use) could obligate reduced heat tolerance. Also, cuticular hydrocarbons (CHCs) reduce evaporative water loss in insects (reviewed in Chown et al., 2011), and cuticular changes due to desiccation can occur quickly in some insects (Bazinet, Marshall, MacMillan, Williams, & Sinclair, 2010). Thus, desiccated insects may allocate resources from other physiological systems (e.g., the heat shock response) to alter CHCs. That said, plasticity in the composition of cuticular hydrocarbons due to desiccation may be limited in ants because CHCs are critical for chemical signaling in this taxon (Martin & Drijfhout, 2009). Related, desiccation may facilitate the allocation of resources from the heat shock response toward other biomolecules associated with desiccation tolerance, such as trehalose, Late Embryonic Abundant proteins, aquaporins, or antioxidants (reviewed in Chown et al., 2011; Thorat & Nath, 2018). Dehydration may also lead to increased catabolism of nutrient reserves (Benoit et al., 2010), which may inhibit an animal's ability to mount a response to heating (Manenti et al., 2018).

Third, desiccation may negatively impact heat tolerance via damage to cellular membranes, inhibition molecular transport, and induction of oxidative stress (reviewed in Alpert, 2006; Minnich, 1982; Toxopeus & Sinclair, 2018). Desiccation‐induced damage may reduce an animal's ability to synthesize or employ protein molecular chaperones (e.g., Hsps) during a heat shock because proteins are also sensitive to desiccation (reviewed in Toxopeus & Sinclair, 2018). In sum, we recommend future work to examine the mechanisms underlying the link between desiccation and reduced heat tolerance given the increasing occurrence of combined of heat and water stress due to ongoing global climate change (Sarhadi et al., 2018).

Previous research on thermal tolerance has shown that non‐native, invasive species may outcompete native species at warmer temperatures (Lejeusne et al., 2014; Rahel, Bierwagen, & Taniguchi, 2008; Zerebecki & Sorte, 2011; but see Verberk et al., 2018). In our study system, native status did not independently influence estimates of heat tolerance (Figure 2a). Estimates of thermal safety margins in our study were consistent with the findings of others across various study systems (e.g., Sunday et al., 2014; reviewed in Rohr et al., 2017) and sensitive to water availability, but our estimates were also not independently influenced by native status. However, our results indicate that native ants may be more reliant on water subsidization in urban environments because *CT*_max_ and estimated thermal safety margins were more sensitive to water availability in native ants relative to non‐native ants. Thus, limited water availability and increasing temperatures may favor non‐native (rather than native) species in some ecosystems, which is important given environments have become increasingly arid and warm (Sarhadi et al., 2018). Future work on additional taxa and more levels of water limitation is required to better understand the complex interplay among native status, urbanization, and water availability related to heat tolerance.

The availability of water, a vital resource for all animals, continues to be put at risk by a combination of increasing temperatures and drier global climates that could leave animals vulnerable due to reduced thermal or hygric safety margins (Burdine & McCluney, 2019; Sarhadi et al., 2018; Sunday et al., 2014). Given the continued natural covariation between elevated temperatures and reduced precipitation (Sarhadi et al., 2018), it is important to continue to consider desiccation resistance as an important physiological metric (Bujan, Yanoviak, & Kaspari, 2016; Burdine & McCluney, 2019; Matzkin, Watts, & Markow, 2007). Our results indicate that water subsidization in urban environments may offset the thermal hazards of the urban heat island effect. However, given urban–rural variation in thermal physiology (Angilletta et al., 2007; Diamond, Chick, Perez, et al., 2017; Pincebourde et al., 2016), similar experimental, comparative studies should be conducted in non‐urban environments where water is not subsidized. We also advocate for examining the role of hydration state in other aspects of thermal sensitivity, such as its effects on thermal optimum or breadth of performance (Angilletta, 2009). In sum, understanding the links between heat tolerance and desiccation resistance will become critical in a world that is increasingly warm, dry, and urbanized.

## CONFLICT OF INTEREST

None declared.

## AUTHOR CONTRIBUTION

**Dustin J. Johnson:** Conceptualization (supporting); Data curation (lead); Investigation (lead); Methodology (lead); Project administration (equal); Writing‐original draft (lead). **Zachary R. Stahlschmidt:** Conceptualization (lead); Data curation (supporting); Formal analysis (lead); Funding acquisition (lead); Investigation (supporting); Methodology (supporting); Project administration (lead); Resources (lead); Software (equal); Supervision (lead); Writing‐original draft (supporting); Writing‐review & editing (lead). 

## Supporting information

Appendix S1

## Data Availability

The datasets supporting this article can be accessed at: https://doi.org/10.6084/m9.figshare.11988918.

## References

[ece36247-bib-0001] Alpert, P. (2006). Constraints of tolerance: Why are desiccation‐tolerant organisms so small or rare? Journal of Experimental Biology, 209, 1575–1584. 16621938 10.1242/jeb.02179

[ece36247-bib-0002] Andrew, N. R. , Hart, R. A. , Jung, M. P. , Hemmings, Z. , & Terblanche, J. S. (2013). Can temperate insects take the heat? A case study of the physiological and behavioural responses in a common ant, Iridomyrmex purpureus (Formicidae), with potential climate change. Journal of Insect Physiology, 59(9), 870–880. 23806604 10.1016/j.jinsphys.2013.06.003

[ece36247-bib-0003] Andrew, N. R. , & Terblanche, J. S. (2013). The response of insects to climate change In Salinger J. (Ed.), Climate of change: Living in a warmer world (pp. 38–50). Auckland, New Zealand: David Bateman Ltd.

[ece36247-bib-0004] Angilletta, M. J. (2009). Thermal adaptation: A theoretical and empirical synthesis. Oxford, UK: Oxford University Press.

[ece36247-bib-0005] Angilletta, M. J. , Wilson, R. S. , Niehaus, A. C. , Sears, M. W. , Navas, C. A. , & Urban, R. P. L. (2007). Physiology: City ants possess high heat tolerance. PLoS ONE, 2(2), e258. 17327918 10.1371/journal.pone.0000258 PMC1797824

[ece36247-bib-0006] AntWeb . (2019). Version 8.3. Available from http://www.antweb.org

[ece36247-bib-0007] Baudier, K. M. , Mudd, A. E. , Erickson, S. C. , & O'Donnell, S. (2015). Microhabitat and body size effects on heat tolerance: Implications for responses to climate change (army ants: Formicidae, Ecitoninae). Journal of Animal Ecology, 84(5), 1322–1330. 26072696 10.1111/1365-2656.12388

[ece36247-bib-0008] Bazinet, A. L. , Marshall, K. E. , MacMillan, H. A. , Williams, C. M. , & Sinclair, B. J. (2010). Rapid changes in desiccation resistance in Drosophila melanogaster are facilitated by changes in cuticular permeability. Journal of Insect Physiology, 56(12), 2006–2012. 20863831 10.1016/j.jinsphys.2010.09.002

[ece36247-bib-0009] Benoit, J. B. , Patrick, K. R. , Desai, K. , Hardesty, J. J. , Krause, T. B. , & Denlinger, D. L. (2010). Repeated bouts of dehydration deplete nutrient reserves and reduce egg production in the mosquito Culex pipiens. Journal of Experimental Biology, 213(16), 2763–2769. 20675546 10.1242/jeb.044883 PMC2912756

[ece36247-bib-0010] Brown, D. A. , Johnson, M. S. , Armstrong, C. J. , Lynch, J. M. , Caruso, N. M. , Ehlers, L. B. , … Moore, R. L. (2007). Short‐term treadmill running in the rat: What kind of stressor is it? Journal of Applied Physiology, 103(6), 1979–1985. 17916671 10.1152/japplphysiol.00706.2007

[ece36247-bib-0011] Bubliy, O. A. , Kristensen, T. N. , Kellermann, V. , & Loeschcke, V. (2012). Plastic responses to four environmental stresses and cross‐resistance in a laboratory population of *Drosophila melanogaster* . Functional Ecology, 26, 245–253.

[ece36247-bib-0012] Buczkowski, G. , & Richmond, D. S. (2012). The effect of urbanization on ant abundance and diversity: A temporal examination of factors affecting biodiversity. PLoS ONE, 7(8), e41729. 22876291 10.1371/journal.pone.0041729 PMC3410901

[ece36247-bib-0013] Bujan, J. , Yanoviak, S. P. , & Kaspari, M. (2016). Desiccation resistance in tropical insects: Causes and mechanisms underlying variability in a Panama ant community. Ecology and Evolution, 6(17), 6282–6291. 27648242 10.1002/ece3.2355 PMC5016648

[ece36247-bib-0014] Burdine, J. D. , & McCluney, K. E. (2019). Differential sensitivity of bees to urbanization‐driven changes in body temperature and water content. Scientific Reports, 9, 1643. 30733542 10.1038/s41598-018-38338-0 PMC6367438

[ece36247-bib-0015] Cahan, S. H. , Nguyen, A. D. , Stanton‐Geddes, J. , Penick, C. A. , Hernaiz‐Hernandez, Y. , DeMarco, B. B. , & Gotelli, N. J. (2017). Modulation of the heat shock response is associated with acclimation to novel temperatures but not adaptation to climatic variation in the ants *Aphaenogaster picea* and *A. rudis* . Comparative Biochemistry and Physiology a‐Molecular & Integrative Physiology, 204, 113–120. 10.1016/j.cbpa.2016.11.017 27894884

[ece36247-bib-0016] Cerda, X. , & Retana, J. (2000). Alternative strategies by thermophilic ants to cope with extreme heat: Individual versus colony level traits. Oikos, 89, 155–163.

[ece36247-bib-0017] Chick, L. D. , Perez, A. , & Diamond, S. E. (2017). Social dimensions of physiological responses to global climate change: What we can learn from ants (Hymenoptera: Formicidae). Myrmecological News, 25, 29–40.

[ece36247-bib-0018] Chown, S. L. , & Duffy, G. A. (2015). Thermal physiology and urbanization: Perspectives on exit, entry and transformation rules. Functional Ecology, 29(7), 902–912.

[ece36247-bib-0019] Chown, S. L. , Sorensen, J. G. , & Terblanche, J. S. (2011). Water loss in insects: An environmental change perspective. Journal of Insect Physiology, 57(8), 1070–1084. 21640726 10.1016/j.jinsphys.2011.05.004

[ece36247-bib-0020] Clemencet, J. , Cournault, L. , Odent, A. , & Doums, C. (2010). Worker thermal tolerance in the thermophilic ant Cataglyphis cursor (Hymenoptera, Formicidae). Insectes Sociaux, 57, 11–15.

[ece36247-bib-0021] Da Lage, J.‐L. , Capy, P. , & David, J.‐R. (1989). Starvation and desiccation tolerance in *Drosophila melanogaster* adults: Effects of environmental temperature. Journal of Insect Physiology, 35, 453–457.

[ece36247-bib-0022] Dahlhoff, E. P. , Buckley, B. A. , & Menge, B. A. (2001). Physiology of the rocky intertidal predator *Nucella ostrina* along an environmental stress gradient. Ecology, 82(10), 2816–2829.

[ece36247-bib-0023] Diamond, S. E. , Chick, L. , Penick, C. A. , Nichols, L. M. , Cahan, S. H. , Dunn, R. R. , … Gotelli, N. J. (2017). Heat tolerance predicts the importance of species interaction effects as the climate changes. Integrative and Comparative Biology, 57(1), 112–120. 28541481 10.1093/icb/icx008

[ece36247-bib-0024] Diamond, S. E. , Chick, L. , Perez, A. , Strickler, S. A. , & Martin, R. A. (2017). Rapid evolution of ant thermal tolerance across an urban‐rural temperature cline. Biological Journal of the Linnean Society, 121(2), 248–257.

[ece36247-bib-0025] Diamond, S. E. , Chick, L. D. , Perez, A. , Strickler, S. A. , & Martin, R. A. (2018). Evolution of thermal tolerance and its fitness consequences: Parallel and non‐parallel responses to urban heat islands across three cities. Proceedings of the Royal Society B: Biological Sciences, 285 10.1098/rspb.2018.0036 PMC6053939 30051828

[ece36247-bib-0026] Duyck, P. F. , David, P. , & Quilici, S. (2006). Climatic niche partitioning following successive invasions by fruit flies in La Reunion. Journal of Animal Ecology, 75(2), 518–526. 16638004 10.1111/j.1365-2656.2006.01072.x

[ece36247-bib-0027] Folguera, G. , Bastias, D. A. , Caers, J. , Rojas, J. M. , Piulachs, M. D. , Belles, X. , & Bozinovic, F. (2011). An experimental test of the role of environmental temperature variability on ectotherm molecular, physiological and life‐history traits: Implications for global warming. Comparative Biochemistry and Physiology a‐Molecular & Integrative Physiology, 159(3), 242–246. 10.1016/j.cbpa.2011.03.002 21406244

[ece36247-bib-0028] Freckleton, R. P. , Harvey, P. H. , & Pagel, M. (2002). Phylogenetic analysis and comparative data: A test and review of evidence. The American Naturalist, 160, 712–726. 10.1086/343873 18707460

[ece36247-bib-0029] Gabriel, W. , Luttbeg, B. , Sih, A. , & Tollrian, R. (2005). Environmental tolerance, heterogeneity, and the evolution of reversible plastic responses. The American Naturalist, 166(3), 339–353. 10.1086/432558 16224689

[ece36247-bib-0030] Gabriel, W. , & Lynch, M. (1992). The selective advantage of reaction norms for environmental tolerance. Journal of Evolutionary Biology, 5(1), 41–59.

[ece36247-bib-0031] Galushko, D. , Ermakov, N. , Karpovski, M. , Palevski, A. , Ishay, J. , & Bergman, D. (2005). Electrical, thermoelectric and thermophysical properties of hornet cuticle. Semiconductor Science and Technology, 20, 286–289.

[ece36247-bib-0032] Gardner, J. L. , Peters, A. , Kearney, M. R. , Joseph, L. , & Heinsohn, R. (2011). Declining body size: A third universal response to warming? Trends in Ecology & Evolution, 26(6), 285–291. 21470708 10.1016/j.tree.2011.03.005

[ece36247-bib-0033] Gaston, K. J. (2009). Geographic range limits of species. Proceedings of the Royal Society B: Biological Sciences, 276(1661), 1391–1393. 10.1098/rspb.2009.0100 PMC2677225 19324808

[ece36247-bib-0034] Gearty, W. , McClain, C. R. , & Payne, J. L. (2018). Energetic tradeoffs control the size distribution of aquatic mammals. Proceedings of the National Academy of Sciences of the United States of America, 115(16), 4194–4199. 29581289 10.1073/pnas.1712629115 PMC5910812

[ece36247-bib-0035] Geerts, A. N. , Vanoverbeke, J. , Vanschoenwinkel, B. , Van Doorslaer, W. , Feuchtmayr, H. , Atkinson, D. , & De Meester, L. (2015). Rapid evolution of thermal tolerance in the water flea Daphnia. Nature Climate Change, 5(10), 665.

[ece36247-bib-0036] Gehring, W. J. , & Wehner, R. (1995). Heat shock protein synthesis and thermotolerance in Cataglyphis, an ant from the Sahara desert. Proceedings of the National Academy of Sciences of the United States of America, 92, 2994–2998. 7708762 10.1073/pnas.92.7.2994 PMC42345

[ece36247-bib-0037] Gotcha, N. , Terblanche, J. S. , & Nyamukondiwa, C. (2018). Plasticity and cross‐tolerance to heterogeneous environments: Divergent stress responses co‐evolved in an African fruit fly. Journal of Evolutionary Biology, 31(1), 98–110. 29080375 10.1111/jeb.13201

[ece36247-bib-0038] Grimm, N. B. , Faeth, S. H. , Golubiewski, N. E. , Redman, C. L. , Wu, J. , Bai, X. , & Briggs, J. M. (2008). Global change and the ecology of cities. Science, 319(5864), 756–760. 18258902 10.1126/science.1150195

[ece36247-bib-0039] Gutierrez‐Pesquera, L. M. , Tejedo, M. , Olalla‐Tarraga, M. A. , Duarte, H. , Nicieza, A. , & Sole, M. (2016). Testing the climate variability hypothesis in thermal tolerance limits of tropical and temperate tadpoles. Journal of Biogeography, 43(6), 1166–1178.

[ece36247-bib-0040] Han, G.‐D. , Cartwright, S. R. , Ganmanee, M. , Chan, B. K. K. , Adzis, K. A. A. , Hutchinson, N. , … Dong, Y.‐W. (2019). High thermal stress responses of Echinolittorina snails at their range edge predict population vulnerability to future warming. Science of the Total Environment, 647, 763–771. 30092533 10.1016/j.scitotenv.2018.08.005

[ece36247-bib-0041] Hansen, T. F. , Pinaar, J. , & Orzack, S. H. (2008). A comparative method for studying adaptation to a randomly evolving environment. Evolution, 62, 1965–1977. 18452574 10.1111/j.1558-5646.2008.00412.x

[ece36247-bib-0042] Harrison, J. F. , Woods, H. A. , & Roberts, S. P. (2012). Ecological and environmental physiology of insects. Oxford, UK: OUP Oxford.

[ece36247-bib-0043] Hemmings, Z. , & Andrew, N. R. (2017). Effects of microclimate and species identity on body temperature and thermal tolerance of ants (Hymenoptera: Formicidae). Austral Entomology, 56(1), 104–114.

[ece36247-bib-0044] Hochachka, P. W. , & Somero, G. N. (2002). Biochemical adaptation. Oxford, UK: Oxford University Press.

[ece36247-bib-0045] Hood, W. G. , & Tschinkel, W. R. (1990). Desiccation resistance in arboreal and terrestrial ants. Physiological Entomology, 15, 23–35.

[ece36247-bib-0046] Horne, C. R. , Hirst, A. G. , & Atkinson, D. (2015). Temperature‐size responses match latitudinal‐size clines in arthropods, revealing critical differences between aquatic and terrestrial species. Ecology Letters, 18(4), 327–335. 25682961 10.1111/ele.12413

[ece36247-bib-0047] Huey, R. B. , & Stevenson, R. D. (1979). Integrating thermal physiology and ecology of ectotherms: A discussion of approaches. Integrative & Comparative Biology, 19, 357–366.

[ece36247-bib-0048] IPCC . (2014). Climate change 2014: Synthesis report. Contribution of Working Groups I, II and III…|Tribal Climate Change Guide [WWW Document]. Retrieved from https://tribalclimateguide.uoregon.edu/literature/ipcc‐2014‐climate‐change‐2014‐synthesis‐report‐contribution‐working‐groups‐i‐ii‐and‐iii

[ece36247-bib-0049] Janda, M. , Folková, D. , & Zrzavý, J. (2004). Phylogeny of Lasius ants based on mitochondrial DNA and morphology, and the evolution of social parasitism in the Lasiini (Hymenoptera: Formicidae). Molecular Phylogenetics and Evolution, 33, 595–614. 15522790 10.1016/j.ympev.2004.07.012

[ece36247-bib-0050] Jumbam, K. R. , Jackson, S. , Terblanche, J. S. , McGeoch, M. A. , & Chown, S. L. (2008). Acclimation effects on critical and lethal thermal limits of workers of the Argentine ant, *Linepithema humile* . Journal of Insect Physiology, 54(6), 1008–1014. 18534612 10.1016/j.jinsphys.2008.03.011

[ece36247-bib-0051] Kaspari, M. (1993). Body size and microclimate use in Neotropical granivorous ants. Oecologia, 96(4), 500–507. 28312456 10.1007/BF00320507

[ece36247-bib-0052] Khaliq, I. , Hof, C. , Prinzinger, R. , Bohning‐Gaese, K. , & Pfenninger, M. (2014). Global variation in thermal tolerances and vulnerability of endotherms to climate change. Proceedings of the Royal Society B: Biological Sciences, 281(1789), 20141097. 10.1098/rspb.2014.1097 PMC4100521 25009066

[ece36247-bib-0053] Kottek, M. , Grieser, J. , Beck, C. , Rudolf, B. , & Rubel, F. (2006). World map of the Koppen‐Geiger climate classification updated. Meteorologische Zeitschrift., 15(3), 259–263.

[ece36247-bib-0054] Le Lagadec, M. D. , Chown, S. L. , & Scholtz, C. H. (1998). Desiccation resistance and water balance in southern African keratin beetles (Coleoptera, Trogidae): The influence of body size and habitat. Journal of Comparative Physiology B‐Biochemical Systemic and Environmental Physiology, 168(2), 112–122.

[ece36247-bib-0055] Leiva, F. P. , Calosi, P. , & Verberk, W. (2019). Scaling of thermal tolerance with body mass and genome size in ectotherms: A comparison between water‐ and air‐breathers. Philosophical Transactions of the Royal Society B: Biological Sciences, 374(1778), 20190035. 10.1098/rstb.2019.0035 PMC6606457 31203753

[ece36247-bib-0056] Lejeusne, C. , Latchere, O. , Petit, N. , Rico, C. , & Green, A. J. (2014). Do invaders always perform better? Comparing the response of native and invasive shrimps to temperature and salinity gradients in southwest Spain. Estuarine Coastal and Shelf Science, 136, 102–111.

[ece36247-bib-0057] Lighton, J. R. B. , & Turner, R. J. (2004). Thermolimit respirometry: An objective assessment of critical thermal maxima in two sympatric desert harvester ants, *Pogonomyrmex rugosus* and *P. californicus* . Journal of Experimental Biology, 207(11), 1903–1913. 15107444 10.1242/jeb.00970

[ece36247-bib-0058] Lutterschmidt, W. I. , & Hutchison, V. H. (1997). The critical thermal maximum: History and critique. Canadian Journal of Zoology‐Revue Canadienne De Zoologie, 75(10), 1561–1574.

[ece36247-bib-0059] Magi, M. , Semchenko, M. , Kalamees, R. , & Zobel, K. (2011). Limited phenotypic plasticity in range‐edge populations: A comparison of co‐occurring populations of two Agrimonia species with different geographical distributions. Plant Biology, 13(1), 177–184. 21143739 10.1111/j.1438-8677.2010.00342.x

[ece36247-bib-0060] Manenti, T. , Cunha, T. R. , Sørensen, J. G. , & Loeschcke, V. (2018). How much starvation, desiccation and oxygen depletion can *Drosophila melanogaster* tolerate before its upper thermal limits are affected? Journal of Insect Physiology, 111, 1–7. 30273554 10.1016/j.jinsphys.2018.09.002

[ece36247-bib-0061] Marron, M. T. , Markow, T. A. , Kain, K. J. , & Gibbs, A. G. (2003). Effects of starvation and desiccation on energy metabolism in desert and mesic Drosophila. Journal of Insect Physiology, 49(3), 261–270. 12770001 10.1016/s0022-1910(02)00287-1

[ece36247-bib-0062] Martin, S. , & Drijfhout, F. (2009). A review of ant cuticular hydrocarbons. Journal of Chemical Ecology, 35(10), 1151–1161. 19866237 10.1007/s10886-009-9695-4

[ece36247-bib-0063] Martinez, B. , Arenas, F. , Trilla, A. , Viejo, R. M. , & Carreno, F. (2015). Combining physiological threshold knowledge to species distribution models is key to improving forecasts of the future niche for macroalgae. Global Change Biology, 21(4), 1422–1433. 24917488 10.1111/gcb.12655

[ece36247-bib-0064] Martins, E. P. , & Hansen, T. F. (1997). Phylogenies and the comparative method: A general approach to incorporating phylogenetic information into the analysis of inter‐specific data. The American Naturalist, 149, 646–667.

[ece36247-bib-0065] Matzkin, L. M. , Watts, T. D. , & Markow, T. A. (2007). Desiccation resistance in four Drosophila species – Sex and population effects. Fly, 1(5), 268–273. 18836314 10.4161/fly.5293

[ece36247-bib-0066] McCarthy, M. P. , Best, M. J. , & Betts, R. A. (2010). Climate change in cities due to global warming and urban effects. Geophysical Research Letters, 37 10.1029/2010GL042845

[ece36247-bib-0067] McCluney, K. E. , Burdine, J. D. , & Frank, S. D. (2017). Variation in arthropod hydration across US cities with distinct climate. Journal of Urban Ecology, 3(1), jux003.

[ece36247-bib-0068] Menke, S. B. , Guenard, B. , Sexton, J. O. , Weiser, M. D. , Dunn, R. R. , & Silverman, J. (2011). Urban areas may serve as habitat and corridors for dry‐adapted, heat tolerant species; an example from ants. Urban Ecosystems, 14(2), 135–163.

[ece36247-bib-0069] Minnich, J. E. (1982). The use of water In Gans C., & Pough F. H. (Eds.), Biology of the reptilia (vol. 12, pp. 325–395). Cambridge, MA: Academic Press.

[ece36247-bib-0070] Mogi, M. , Miyagi, I. , Abadi, K. , & Syafruddin, K. (1996). Inter‐ and intraspecific variation in resistance to desiccation by adult Aedes (Stegomyia) spp (Diptera: Culicidae) from Indonesia. Journal of Medical Entomology, 33(1), 53–57. 8906905 10.1093/jmedent/33.1.53

[ece36247-bib-0071] Moreau, C. S. , Bell, C. D. , Vila, R. , Archibald, S. B. , & Pierce, N. E. (2006). Phylogeny of the ants: Diversification in the age of angiosperms. Science, 312, 101–104. 16601190 10.1126/science.1124891

[ece36247-bib-0072] Moreno, J. , Merino, S. , Martinez, J. , Sanz, J. J. , & Arriero, E. (2002). Heterophil/lymphocyte ratios and heat‐shock protein levels are related to growth in nestling birds. Ecoscience, 9(4), 434–439.

[ece36247-bib-0073] Nguyen, A. D. , DeNovellis, K. , Resendez, S. , Pustilnik, J. D. , Gotelli, N. J. , Parker, J. D. , & Cahan, S. H. (2017). Effects of desiccation and starvation on thermal tolerance and the heat‐shock response in forest ants. Journal of Comparative Physiology B: Biochemical Systems and Environmental Physiology, 187(8), 1107–1116. 10.1007/s00360-017-1101-x 28439669

[ece36247-bib-0074] Oke, T. R. (1973). City size and the urban heat island. Atmospheric Environment, 7(8), 769–779.

[ece36247-bib-0075] Oreskes, N. (2004). The scientific consensus on climate change. Science, 306(5702), 1686. 15576594 10.1126/science.1103618

[ece36247-bib-0076] Overgaard, J. , Kristensen, T. N. , & Sørensen, J. G. (2012). Validity of thermal ramping assays used to assess thermal tolerance in arthropods. PLoS ONE, 7, e32758. 22427876 10.1371/journal.pone.0032758 PMC3302897

[ece36247-bib-0077] Penick, C. A. , Savage, A. M. , & Dunn, R. R. (2015). Stable isotopes reveal links between human food inputs and urban ant diets. Proceedings of the Royal Society B: Biological Sciences, 282(1806), 20142608. 10.1098/rspb.2014.2608 PMC4426608 25833850

[ece36247-bib-0078] Peters, R. H. (1986). The ecological implications of body size. Cambridge, UK: Cambridge University Press.

[ece36247-bib-0079] Pincebourde, S. , & Casas, J. (2019). Narrow safety margin in the phyllosphere during thermal extremes. Proceedings of the National Academy of Sciences of the United States of America, 116(12), 5588–5596. 30782803 10.1073/pnas.1815828116 PMC6431205

[ece36247-bib-0080] Pincebourde, S. , Murdock, C. C. , Vickers, M. , & Sears, M. W. (2016). Fine‐scale microclimatic variation can shape the responses of organisms to global change in both natural and urban environments. Integrative and Comparative Biology, 56(1), 45–61. 27107292 10.1093/icb/icw016

[ece36247-bib-0081] Pincebourde, S. , & Suppo, C. (2016). The vulnerability of tropical ectotherms to warming is modulated by the microclimatic heterogeneity. Integrative and Comparative Biology, 56(1), 85–97. 27371561 10.1093/icb/icw014

[ece36247-bib-0082] Rahel, F. J. , Bierwagen, B. , & Taniguchi, Y. (2008). Managing aquatic species of conservation concern in the face of climate change and invasive species. Conservation Biology, 22(3), 551–561. 18577084 10.1111/j.1523-1739.2008.00953.x

[ece36247-bib-0083] Raubenheimer, D. , & Gade, G. (1994). Hunger‐thirst interactions in the Locust, *Locusta migratoria* . Journal of Insect Physiology, 40(7), 631–639.

[ece36247-bib-0084] Raubenheimer, D. , & Gade, G. (1996). Separating food and water deprivation in locusts: Effects on the patterns of consumption, locomotion and growth. Physiological Entomology, 21(1), 76–84.

[ece36247-bib-0085] Rezende, E. L. , Bozinovic, F. , & Garland, T. (2004). Climatic adaptation and the evolution of basal and maximum rates of metabolism in rodents. Evolution, 58(6), 1361–1374. 15266984 10.1111/j.0014-3820.2004.tb01714.x

[ece36247-bib-0086] Ribeiro, P. L. , Camacho, A. , & Navas, C. A. (2012). Considerations for assessing maximum critical temperatures in small ectothermic animals: Insights from leaf‐cutting ants. PLoS ONE, 7, 1–7. 10.1371/journal.pone.0032083 PMC3286443 22384147

[ece36247-bib-0087] Rohr, J. R. , Civitello, D. J. , Cohen, J. M. , Roznik, E. A. , Sinervo, B. , & Dell, A. I. (2017). A global framework for estimating acclimation and thermal breadth predicts risk from climate change. bioRxiv.

[ece36247-bib-0088] Sammut, I. A. , & Harrison, J. C. (2003). Cardiac mitochondrial complex activity is enhanced by heat shock proteins. Clinical and Experimental Pharmacology and Physiology, 30(1–2), 110–115. 12542463 10.1046/j.1440-1681.2003.03799.x

[ece36247-bib-0089] Sanders, N. J. , Barton, K. E. , & Gordon, D. M. (2001). Long‐term dynamics of the distribution of the invasive Argentine ant, Linepithema humile, and native ant taxa in northern California. Oecologia, 127(1), 123–130. 28547163 10.1007/s004420000572

[ece36247-bib-0090] Sarhadi, A. , Ausín, M. C. , Wiper, M. P. , Touma, D. , & Diffenbaugh, N. S. (2018). Multidimensional risk in a nonstationary climate: Joint probability of increasingly severe warm and dry conditions. Science Advances, 4(11), eaau3487. 30498780 10.1126/sciadv.aau3487 PMC6261656

[ece36247-bib-0091] Savage, V. M. , Gillooly, J. F. , Brown, J. H. , West, G. B. , & Charnov, E. L. (2004). Effects of body size and temperature on population growth. The American Naturalist, 163(3), 429–441. 10.1086/381872 15026978

[ece36247-bib-0092] Scheffers, B. R. , Edwards, D. P. , Diesmos, A. , Williams, S. E. , & Evans, T. A. (2014). Microhabitats reduce animal's exposure to climate extremes. Global Change Biology, 20(2), 495–503. 24132984 10.1111/gcb.12439

[ece36247-bib-0093] Schilman, P. E. , Lighton, J. R. B. , & Holway, D. A. (2007). Water balance in the Argentine ant (*Linepithema humile*) compared with five common native ant species from southern California. Physiological Entomology, 32(1), 1–7.

[ece36247-bib-0094] Sexton, J. P. , McIntyre, P. J. , Angert, A. L. , & Rice, K. J. (2009). Evolution and ecology of species range limits. Annual Review of Ecology Evolution and Systematics, 40, 415–436.

[ece36247-bib-0095] Sinclair, B. J. , Marshall, K. E. , Sewell, M. A. , Levesque, D. L. , Willett, C. S. , Slotsbo, S. , … Huey, R. B. (2016). Can we predict ectotherm responses to climate change using thermal performance curves and body temperatures? Ecology Letters, 19(11), 1372–1385. 27667778 10.1111/ele.12686

[ece36247-bib-0096] Smit, B. , Whitfield, M. C. , Talbot, W. A. , Gerson, A. R. , McKechnie, A. E. , & Wolf, B. O. (2018). Avian thermoregulation in the heat: Phylogenetic variation among avian orders in evaporative cooling capacity and heat tolerance. The Journal of Experimental Biology, 221(6), jeb174870. 29440359 10.1242/jeb.174870

[ece36247-bib-0097] Smith, F. A. , & Lyons, S. K. (2013). Animal body size: Linking pattern and process across space, time, and taxonomic group. Chicago, IL: University of Chicago Press.

[ece36247-bib-0098] Stahlschmidt, Z. R. , & Johnson, D. (2018). Moving targets: Determinants of nutritional preferences and habitat use in an urban ant community. Urban Ecosystems, 21(6), 1151–1158.

[ece36247-bib-0099] Stillman, J. H. , & Somero, G. N. (2000). A comparative analysis of the upper thermal tolerance limits of eastern Pacific porcelain crabs, genus Petrolisthes: Influences of latitude, vertical zonation, acclimation, and phylogeny. Physiological and Biochemical Zoology, 73(2), 200–208. 10801398 10.1086/316738

[ece36247-bib-0100] Sunday, J. M. , Bates, A. E. , Kearney, M. R. , Colwell, R. K. , Dulvy, N. K. , Longino, J. T. , & Huey, R. B. (2014). Thermal‐safety margins and the necessity of thermoregulatory behavior across latitude and elevation. Proceedings of the National Academy of Sciences of the United States of America, 111(15), 5610–5615. 24616528 10.1073/pnas.1316145111 PMC3992687

[ece36247-bib-0101] Thorat, L. , & Nath, B. B. (2018). Insects with survival kits for desiccation tolerance under extreme water deficits. Frontiers in Physiology, 9, 1843. 30622480 10.3389/fphys.2018.01843 PMC6308239

[ece36247-bib-0102] Toxopeus, J. , & Sinclair, B. J. (2018). Mechanisms underlying insect freeze tolerance. Biological Reviews, 93(4), 1891–1914. 10.1111/brv.1242 29749114

[ece36247-bib-0103] Underwood, E. C. , & Fisher, B. L. (2006). The role of ants in conservation monitoring: If, when, and how. Biological Conservation, 132(2), 166–182.

[ece36247-bib-0104] Vahmani, P. , & Jones, A. D. (2017). Water conservation benefits of urban heat mitigation. Nature Communications, 8(1). 10.1038/s41467-017-01346-1 PMC5651875 29057940

[ece36247-bib-0105] Verberk, W. , Leuven, R. , van der Velde, G. , & Gabel, F. (2018). Thermal limits in native and alien freshwater peracarid Crustacea: The role of habitat use and oxygen limitation. Functional Ecology, 32(4), 926–936. 29937614 10.1111/1365-2435.13050 PMC5993316

[ece36247-bib-0106] Vonshak, M. , & Gordon, D. (2015). Intermediate disturbance promotes invasive ant abundance. Biological Conservation, 186, 359–367.

[ece36247-bib-0107] Ward, P. S. , Brady, S. G. , Fisher, B. L. , & Schultz, T. R. (2015). The evolution of myrmicine ants: Phylogeny and biogeography of a hyperdiverse ant clade (Hymenoptera: Formicidae). Systematic Entomology, 40, 61–81.

[ece36247-bib-0108] Wiens, J. J. , Graham, C. H. , Moen, D. S. , Smith, S. A. , & Reeder, T. W. (2006). Evolutionary and ecological causes of the latitudinal diversity gradient in hylid frogs: Treefrog trees unearth the roots of high tropical diversity. The American Naturalist, 168(5), 579–596. 10.1086/507882 17080358

[ece36247-bib-0109] Youngsteadt, E. , Dale, A. G. , Terando, A. J. , Dunn, R. R. , & Frank, S. D. (2015). Do cities simulate climate change? A comparison of herbivore response to urban and global warming. Global Change Biology, 21(1), 97–105. 25163424 10.1111/gcb.12692

[ece36247-bib-0110] Zerebecki, R. A. , & Sorte, C. J. B. (2011). Temperature tolerance and stress proteins as mechanisms of invasive species success. PLoS ONE, 6(4), e14806. 21541309 10.1371/journal.pone.0014806 PMC3082523

[ece36247-bib-0111] Zhang, Y. Y. , & Kieffer, J. D. (2014). Critical thermal maximum (CTmax) and hematology of shortnose sturgeons (*Acipenser brevirostrum*) acclimated to three temperatures. Canadian Journal of Zoology, 92(3), 215–221.

