# Supplementary material for: City limits: Heat tolerance is influenced by body size and hydration state in an urban ant community
Source: Ecol Evol. 2020 Apr 15;10(11):4944–55. doi: 10.1002/ece3.6247 (PMC7297767; doi:10.1002/ece3.6247)
Supplement: Supplementary file 1 — Appendix S1 [file ECE3-10-4944-s001.docx]

**Supporting Information**

*Estimating Effect of Water Availability on Evaporative Cooling Potential*

Hydration may confer greater evaporative cooling potential because greater water stores can be deployed (i.e., lost) during periods of heat stress. We indirectly assessed this mechanism in our study by first converting the average difference in absolute water content (mg of H_2_O / individual) between the two water treatment groups to energy. For example, after 32 h of treatment, the average water-unlimited *F. moki* contained 1.5 mg more water than the average water-limited *F. moki*, and this water difference translates to water-unlimited *F. moki* having an absolute maximum of 0.0062 J of additional evaporative cooling due to the specific heat capacity of water (4.184 J g°C^-1^). Next, we used the following equation to solve for the temperature difference (i.e., cooling) resulting from this amount of evaporative heat loss.

, where is the change of energy (J; e.g., 0.0062 J, see above), is the specific heat capacity of insects (3.3472 J g°C^-1^: Shinozaki, 1957), M is the mass of the animal (average water-unlimited *F. moki* live mass: 4.9 mg), and is the change in temperature.


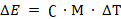

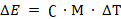

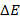

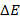

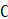

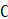

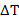

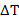


Using this approach, we estimated the evaporative cooling advantage of hydration to result in <0.4°C in *F. moki*—yet, the average CT_max_ difference between water-unlimited and water-limited *F. moki* was 3.1°C after 32 h of treatment (Fig. 3B). Note: *F. moki* at 32 h of treatment was used in this example because of the large difference in absolute water content between the two treatment groups. Thus, water treatment-dependent evaporative cooling potential would be relatively high in this case—in contrast, for example, we estimate that water supplementation for *P. imparis* at 32 h of treatment resulted in <0.1°C of additional evaporative cooling. Together, these calculations reveal that hydration likely did not confer an appreciable evaporative cooling advantage of animals in our study given significant differences in CT_max_ between water-limited and -unlimited animals typically ranged from 1 – 3°C.

Shinozaki, J., 1957. The Specific Heat of Insects. J. Fac. Sci. HOKKAIDO Univ. Ser. VI Zool. 6.

*Supporting Figure*

**Figure S1.** Phylogenetic tree demonstrating the relative relationships among ant species, using estimated minimum branch lengths, in Experiment 1 (see Supporting Information: *Phylogenetic Methods* above). Only *P. imparis* and *F. moki* were used in Experiment 2. Native species’ names are bolded, non-native species’ names are not bolded, and the time scale is denoted (Mya).


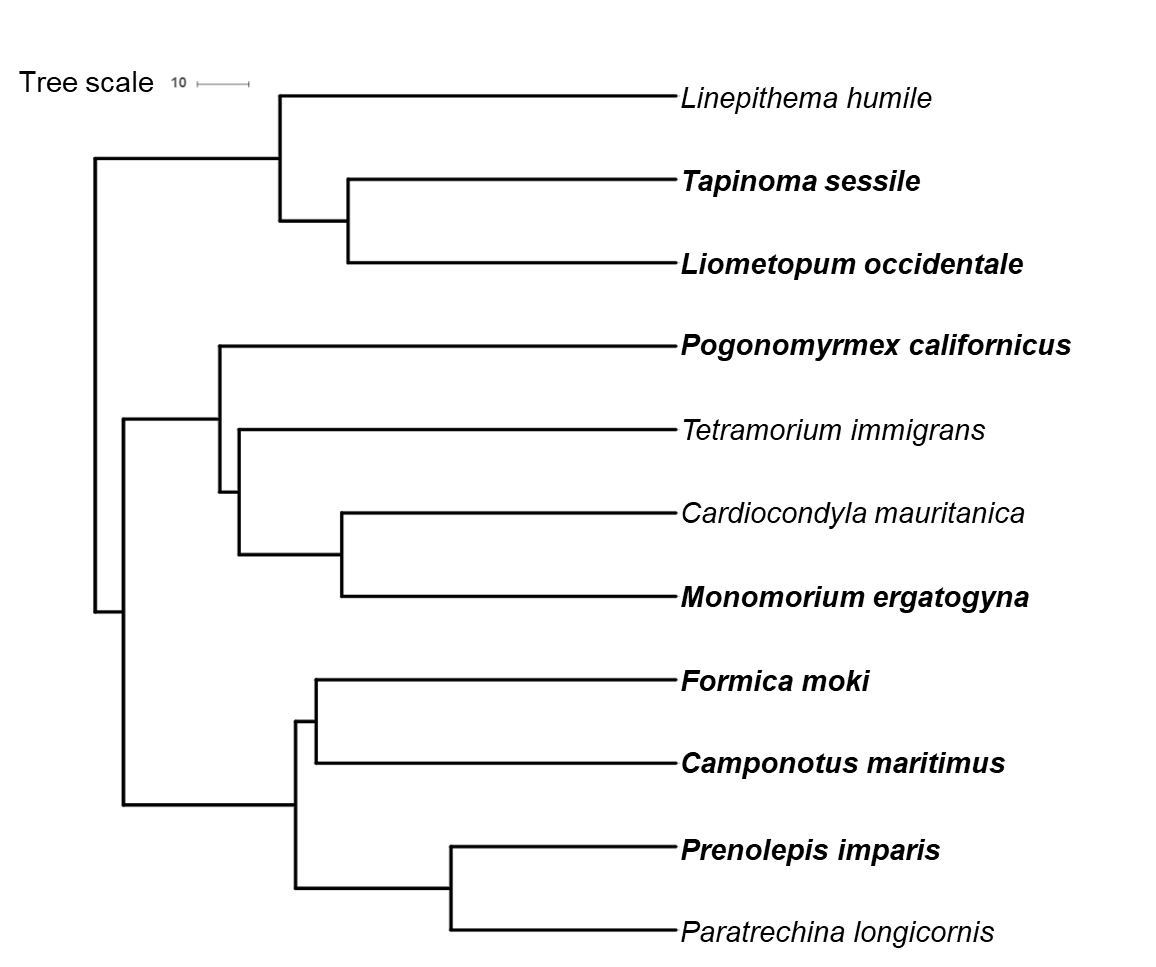


*Supporting Table*

**Table S1.** Number of colonies from which ants were collected, number of individuals in each group replicate that were housed in lab from each colony after collection, and the number of group replicates per species in Experiment 1. Native species’ names are bolded, and non-native species’ names are not bolded.

| **Species** | **# of colonies** | **# of individuals per group replicate** | **# of group replicates** |
| --- | --- | --- | --- |
| *Linepithema humile* | 4 | 15 | 16 |
| ***Tapinoma sessile*** | 2 | 15 | 8 |
| ***Liometopum occidentale*** | 4 | 15 | 20 |
| ***Pogonomyrmex californicus*** | 4 | 15 | 12 |
| *Tetramorium immigrans* | 6 | 15 | 20 |
| *Cardiocondyla mauritanica* | 1 | 15 | 4 |
| ***Monomorium ergatogyna*** | 3 | 30 | 6 |
| ***Formica moki*** | 2 | 15 | 10 |
| ***Camponotus maritimus*** | 1 | 1 | 2 |
| ***Prenolepis imparis*** | 8 | 15 | 28 |
| *Paratrechina longicornis* | 2 | 15 | 8 |
